# Are pigs overestimated as a source of zoonotic influenza viruses?

**DOI:** 10.1186/s40813-022-00274-x

**Published:** 2022-06-30

**Authors:** Christin Hennig, Annika Graaf, Philipp P. Petric, Laura Graf, Martin Schwemmle, Martin Beer, Timm Harder

**Affiliations:** 1 Institute of Diagnostic Virology, Friedrich-Loeffler-Institut, Suedufer 10, 17493 Greifswald-Insel Riems, Germany; 2 Institute of Virology, Medical Center, University of Freiburg, 79104 Freiburg, Germany; 3 Faculty of Medicine, University of Freiburg, 79104 Freiburg, Germany; 4 Spemann Graduate School of Biology and Medicine, University of Freiburg, 79104 Freiburg, Germany

**Keywords:** Swine influenza A virus, Mixing vessel, Zoonotic potential, Reverse zoonosis, Surveillance

## Abstract

**Background:**

Swine influenza caused by influenza A viruses (IAV) directly affects respiratory health and indirectly impairs reproduction rates in pigs causing production losses. In Europe, and elsewhere, production systems have intensified featuring fewer holdings but, in turn, increased breeding herd and litter sizes. This seems to foster swine IAV (swIAV) infections with respect to the entrenchment within and spread between holdings. Disease management of swine influenza is difficult and relies on biosecurity and vaccination measures. Recently discovered and widely proliferating forms of self-sustaining modes of swIAV infections in large swine holdings challenge these preventive concepts by generating vaccine-escape mutants in rolling circles of infection.

**Main body:**

The most recent human IAV pandemic of 2009 rooted at least partly in IAV of porcine origin highlighting the zoonotic potential of swIAV. Pigs constitute a mixing vessel of IAV from different species including avian and human hosts. However, other host species such as turkey and quail but also humans themselves may also act in this way; thus, pigs are not essentially required for the generation of IAV reassortants with a multispecies origin. Since 1918, all human pandemic influenza viruses except the H2N2 virus of 1958 have been transmitted in a reverse zoonotic mode from human into swine populations. Swine populations act as long-term reservoirs of these viruses. Human-derived IAV constitute a major driver of swIAV epidemiology in pigs. Swine-to-human IAV transmissions occurred rarely and mainly sporadically as compared to avian-to-human spill-over events of avian IAV. Yet, new swIAV variants that harbor zoonotic components continue to be detected. This increases the risk that such components might eventually reassort into viruses with pandemic potential.

**Conclusions:**

Domestic pig populations should not be globally stigmatized as the only or most important reservoir of potentially zoonotic IAV. The likely emergence from swine of the most recent human IAV pandemic in 2009, however, emphasized the principal risks of swine populations in which IAV circulate unimpededly. Implementation of regular and close-meshed IAV surveillance of domestic swine populations to follow the dynamics of swIAV evolution is clearly demanded. Improved algorithms for directly inferring zoonotic potential from whole IAV genome sequences as well as improved vaccines are still being sought.

## Background

Despite the current dominance of SARS coronavirus-2, influenza A viruses (IAV) remain an imminent global threat to public health and even more so for livestock welfare worldwide [1, 2]. Due to the segmented nature of their RNA genome and their error-prone RNA replication machinery, IAV are genetically highly flexible and may adapt rapidly by genetic drift and genetic shift to new hosts [3]. Hence, IAV in both avian and mammalian host species are capable of evading innate as well as natural and vaccine-induced adaptive immunity of their host populations and of overcoming species barriers [1, 2].

Swine influenza A viruses (swIAV) of the subtypes H1N1, H1N2 and H3N2 co-circulate globally and seasonally independently causing respiratory disease and indirectly reproductive losses in pigs. Thereby, swIAV compromises animal welfare and invokes economic damage in the pig industry [1, 4]. In addition, swine populations have been the source of generating human pandemic IAV as demonstrated in 2009 when a new reassortant IAV of the H1N1 subtype emerged in pigs in Mesoamerica [5]. This virus harbored gene segments derived from human, avian and porcine origin. Pigs have previously been proposed to act as a “mixing vessel” for IAV of different host origins. Co-infections in pigs with IAV of porcine, human or avian origin can generate novel reassortant swIAV, bearing zoonotic or even pandemic potential [6–8]. This is partially based on the presence, high density and distribution pattern of the two viral entry receptors, used by avian and mammalian IAV, in the porcine respiratory tract [9–11].

The majority of sporadically reported, natural infections of pigs with avian and most human seasonal IAV has not succeeded in building stable lineages that independently circulate in the swine population, although such spill-over events may occur more frequently than previously thought [2, 6, 12]. Nevertheless, reverse zoonotic transmissions of some IAV from humans into pig populations had a major impact on the establishment of IAV lines that circulate in pigs since decades: Historically, the first of these lines, H1N1 (classical, 1A according to the most recent nomenclature [13]), was transmitted in the wake of the 1918 Spanish flu, the first well-documented human pandemic associated with a high case-fatality rate in the human population in the twentieth century [14–16]. Three additional human IAV pandemics were noted in the past century, whereof two of these viruses also ended up in pigs, the H3N2 virus of the 1968 "Hong Kong flu" and the H1N1 virus (seasonal, 1B) of the so-called “Russian flu” in 1977. The sole exception seems to be the H2N2 pandemic virus of the “Asian flu” of 1958. To date there is a single avian lineage, H1N1 (H1 avian-like/H1av or 1C), that has established stable circulation in the European and in parts of the Asian pig population since the late 1970s [17–20].

## Zoonotic swIAV infections are reported regularly but cases mainly remain sporadic

An ever-increasing intensification of pig production worldwide and the growing cross-border trade, also in live pigs, acts to expand the interface between pigs and humans. The industrialization of livestock production may create new reservoirs of IAV and favor reciprocal IAV transmissions between species [21–24]. Zoonotic interspecies transmission of IAV at the swine-human interface usually requires an exposure of a highly susceptible individual to a high virus load. Such occasions are potentially enabled for example at agricultural fairs, live animal markets or in swine holdings. In general, close contact to swine raises the risk for human infections with swIAV [14, 25]. Two cohort studies examining antibodies against swine H1N1 [21, 23] and swine H3N2 IAV showed significantly higher antibody titers in swine workers compared to the general public suggesting an increased occupational risk of swIAV infection [21]. It should be noted, however, that serological cross-reactions with human IAV antigens frequently interfere with result interpretation of such studies. Detection of replicating swIAV in human hosts, in contrast, clearly proves infection. Sporadic zoonotic IAV infections originating from pigs are regularly detected (Table 1). In the majority of cases, only individual humans are affected. Rarely, clustered outbreaks were reported, which were caused rather by a common source of infection (e.g., pig fairs and shows in the US [26–30]) than by efficient human-to-human transmission. The establishment of stably circulating lineages in humans from such events has been extremely rare. As already mentioned, an important exception is the most recent human pandemic virus H1N1pdm09, whose origin has been narrowed down to pig populations in Mesoamerica [31, 32].

**Table 1:** Human infections with influenza A viruses of porcine origin

Continent	Country	Subtype	Year	Cases*	Subtype	References
North America	United States	A(H3N2)v	2010/11	7	n.d	[33]
			2012	315 (283, 2 ic)	306 TRIG; M H1N1pdm09; 9 n.d	[34, 33]
			2012/13	20	n.d	[33]
			2013/14	3	n.d	[33]
			2015	3 (1 ic)	n.d	[35, 36]
			2016	18 (16)	H3hu	[27, 29, 37]
			2017	62 (37)	H3hu	[28, 38, 39]
			2018	2 (1)	n.d	[33, 40]
			2020	1	n.d	[41]
			2021	2 (1)	1 H3hu; 1 n.d	[33, 42]
			2021/22	1	n.d	[33]
		A(H1N1)v	2011/12	2	n.d	[33]
			2012/13	2	n.d	[33]
			2015	3	n.d	[35]
			2015/16	1	n.d	[33]
			2017	1	H1N1pdm09	[43, 38, 44]
			2019	1 ic	H1N1pdm09	[43, 45, 46]
			2020/21	8	1 H1N1pdm09; 7 n.d	[43, 33, 41, 47]
		A(H1N2)v	2011/12	4	n.d	[33, 47]
			2015/16	3	n.d	[37]
			2017	4 (3)	n.d	[38, 48, 49]
			2018	14 (12)	n.d	[40, 50]
			2020/21	4	n.d	[33]
			2021/22	1	n.d	[33]
	Canada	A(H3N2)v	2016	1	n.d	[37]
		A(H1N2)v	2020	1 (1)	n.d	[45, 51]
South America	Brazil	A(H1N2)v	2015	1	n.d	[35, 52]
			2020	2 (1)	n.d	[45, 53]
Europe	Germany	A(H1N1)v	2010	1 ic	H1avN1	[54]
			2011	1 (1)	H1avN1	[54]
			2020	1 (1)	H1avN1	[45, 53]
			2021	1 (1)	H1avN1	[55]
		A(H1N2)v	2011	1 (1)	H1huN2	[54]
	Italy	A(H1N1)v	2016	1	H1avN1	[37, 56]
	Switzerland	A(H1N1)v	2016	1	H1avN1	[37, 56]
			2017	1	H1avN1	[38, 39]
	Netherlands	A(H1N1)v	2016	1 (1)	H1avN1	[37]
			2019	1	H1avN1	[57]
			2020	1 ic	H1avN1	[42]
	France	A(H1N1)v	2018	1	H1N1pdm09	[58]
Asia	China	A(H1N1)v	2012	1 (1)	H1avN1	[59]
			2015	1 (1)	H1avN1	[60]
			2016	4 (3)	H1avN1	[61, 62]
			2019	1	H1avN1	[40]
			2020	5 (5)	H1avN1	[42, 45]
			2021	6	n.d	[63]
Australia		A(H3N2)v	2018	1	n.d	[40]
			2019	1 (1)	n.d	[64]
			2021	1 (1)	H3hu	[47]

*Numbers in brackets refer to patients younger than 18 years; v: variant; ic: immunocompromised person

n.d.—Not defined

The first major outbreak of swIAV in a human population dates back to 1976 and affected recruits in a military base in Fort Dix, New Jersey, US: A total of 230 soldiers contracted swIAV of the H1N1 subtype, including one fatal case. The virus was introduced after the winter holiday season and spread rapidly within one unit. However, further human-to-human transmission outside the training group was limited. It still remains unknown how the virus entered the base and why it did not spread beyond Fort Dix, as no soldier stated previous contact to swine and no corresponding case outside the military base was reported [65]. Apart from this event, between 1958 and 2009, 73 isolated swIAV cases in humans were reported worldwide with a case fatality rate of 10% [66, 67]. In April 2009, first infections with a novel H1N1 swIAV were described in children in the US. Within two months, several ten thousand cases in 74 countries had been reported, confirming the high contagiosity of this virus. The genetic constellation of this novel virus consisted of gene segments from avian, swine and human origin [8, 14]. The 2009 pandemic strain rapidly re-entered the swine population via reverse-zoonotic transmissions, which have been detected frequently, worldwide, and are continuing up to this date [18]. As a consequence, reassortment events with circulating authentic swIAV strains have increased genetic diversity which may favor the emergence of novel reassortant swIAV with enhanced zoonotic potential [68]. However, timely detection of such strains and their proper risk evaluation remain challenging even to date. Detection of swine-origin H1N1pdm09 in the human population would require full genome sequencing and species-specific mutation pattern definition [43].

Among such novel swIAV “v”ariants (flagged with a “v” to indicate the swine origin) H3N2v caused clustered, local outbreaks of zoonotic influenza in North America. In 2012, 306 cases of infection were reported after direct or indirect exposure to (asymptomatically) infected swine (Table 1). All “variant” viruses harbored the matrix (M) gene segment derived from the pandemic H1N1pdm09. In experiments in pigs, the M segment has been identified as a determinant of respiratory transmission efficiency. In addition, a combination of the neuraminidase (NA) and M genes of H1N1pdm09 was found essential to facilitate efficient transmission and replication in pigs [69]. Initial concerns of a higher human-to-human transmission rate through the H1N1pdm09 derived M gene proved to be unjustified though [34, 70, 71]. Further clustered zoonotic transmission events occurred in the United States and were related to agricultural fairs and live animal markets with severe incidences in 2016 and 2017 [28, 29]. To date, a total of 483 cases of novel swIAV infections in humans have been reported to the Centers of Disease Control and Prevention in the United States since 2010, including not only infections with H3N2v, but also with H1N1v and H1N2v [33, 72].

In China, recently a new genotype (referred to as G4) emerged and gained predominance in swine populations since 2016. G4 is a reassortant Eurasian avian-like H1N1 virus, which contains 2009 pandemic and triple-reassortant derived internal genes [61]. It preferentially binds to human-type receptors and was claimed to bear the potential to transmit efficiently between humans, although evidence was based on serological data alone as no productive virus infections in humans have been reported to date [59, 61, 73].

In Europe, cases of swIAV infections have been documented in a variety of countries affecting mainly swine farmers, staff of swine holdings or their (younger) family members. Most patients showed influenza-like symptoms and the infections run a benign course [57, 58]. In Germany, between 2007 and 2021, several swIAV cases were reported, affecting mostly children, teens and one immunocompromised adult [74]. The majority of human infections in Europe was caused by the Eurasian avian-like H1N1 swIAV which is the most prominent subtype in European pig populations [18]. This subtype also shows the largest antigenic distance to the H1 IAV circulating in the human population [75]. Although, the surveillance of swIAV has intensified since 2009, it cannot be excluded that the true number of cases of human swIAV infections is higher than suggested by the low number of reported cases, as symptoms in humans are indistinguishable from seasonal influenza [66]. Since swIAV are circulating year-round in swine populations, presentation of flu-like symptoms in patients outside the human influenza season of a certain region combined with a history of occupational contact to pigs should raise suspicion justifying virological examination of such cases.

## The pig is not an exclusive “mixing vessel” for IAV

The mixing vessel hypothesis was coined by Scholtissek et al. They defined pigs as a reassortant machine for IAV of various host origins [76]. This concept builds on the susceptibility of pigs to various IAV from mammalian as well as avian sources. Depending on the species origin, these viruses have distinct predilections for sialic acid (SA) receptors of the SA α2-6Gal (human-adapted) or the SA α2-3Gal type (avian-adapted). Presence of both receptor types in the respiratory tract of pigs is a prerequisite for their function as a “mixing vessel”. In line with this hypothesis and despite the gross dominance of SA α2-6 receptors, especially in the upper respiratory tract of pigs, as shown by virus binding studies, lectin histochemistry and enzymatic analyses, porcine-adapted IAV often retain binding affinity to both receptor types [9, 77–79]. Switches in receptor binding efficacy is regulated by very few amino acids in the receptor binding unit of the viral hemagglutinin (HA) attachment protein. In particular, positions 190 and 225 impact receptor specificity [2].

Recent findings from studies investigating the role of host factors in restricting the host range of IAV further support the mixing vessel hypothesis. The viral polymerase requires the presence of the cellular factor Acidic Nuclear Phosphoprotein 32 Family Member A (ANP32A) for its activity. Mammalian ANP32A proteins, however, do not support efficient polymerase activity of avian IAV necessitating adaptive mutations in the viral polymerase of avian IAV for successful replication in a mammalian host when jumping the species barrier [80]. Interestingly, swine ANP32A is the exception among mammalian ANP32A proteins because it supports avian IAV polymerase activity to some extent [81, 82] which might further explain the susceptibility of pigs to avian IAV.

**Fig. 1:**
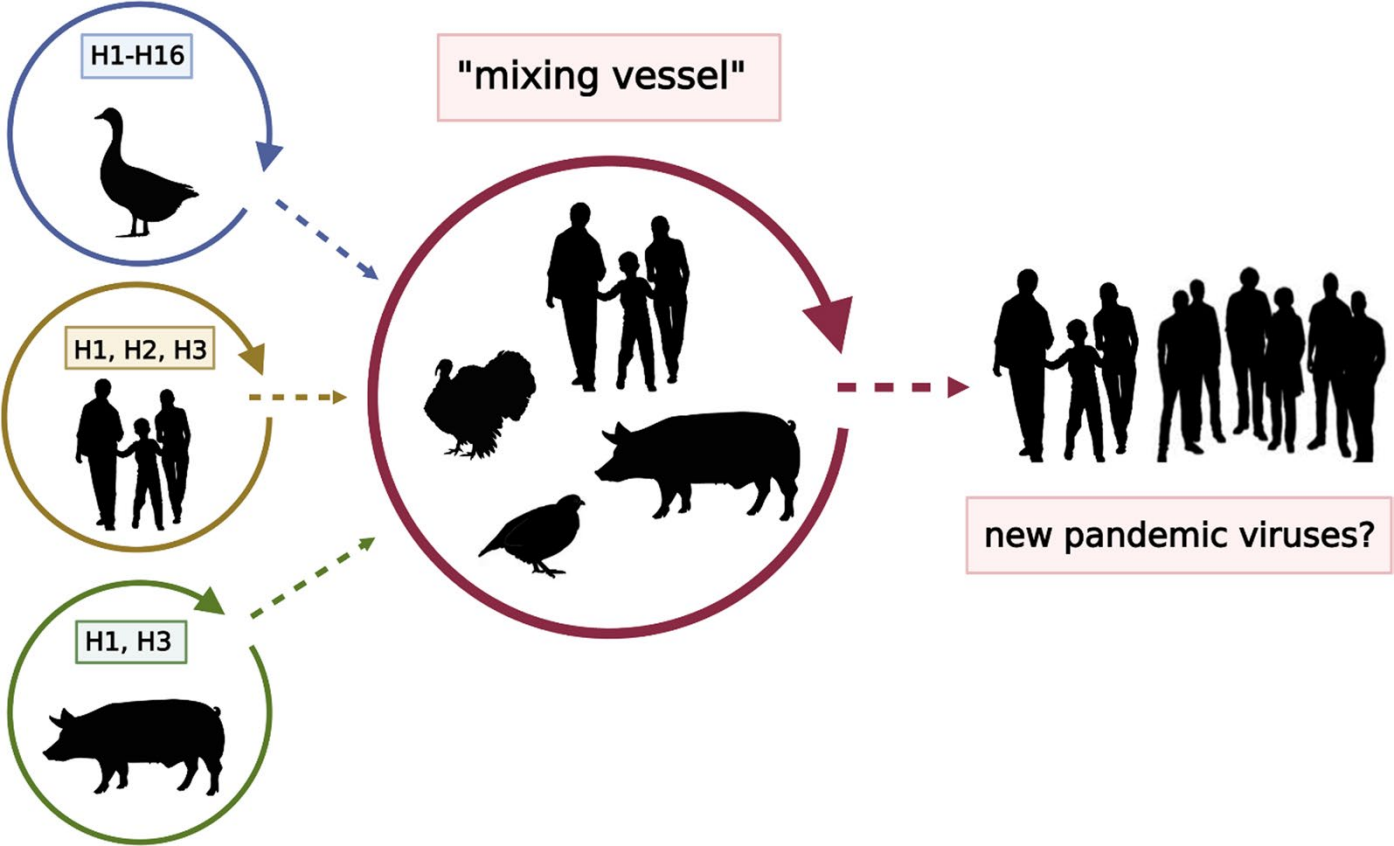
Schematic presentation of putative “mixing vessel” host species (pigs, quails, turkeys, humans) which express sialic acid receptors for both avian- and human-adapted influenza A viruses (IAV) in their respiratory tracts. Hence, they are considered susceptible for a wider range of IAV of different host origins. Co-infections with different IAV create reassortment opportunities increasing the likelihood of the formation of reassortants with increased zoonotic or pre-pandemic propensity

The initial assumption of Scholtissek et al. that swine are essentially required to generate reassortants between avian and mammalian IAV, however, has been challenged as both receptor types have also been detected in humans, quails and other avian species, particularly, in turkeys [10, 83, 84] (Fig. 1). While the receptor distribution in tissues and their densities at the cell surface differ grossly between those species, they resemble each other closely in the human and porcine respiratory systems [85, 86]. Likewise, different isoforms of ANP32A in several avian species facilitate a more mammalian-like adaptation of the IAV polymerase in these birds, further challenging the necessity of pigs as a unique mixing vessel [87].

It should be noted that there is no evidence for the participation of swIAV-derived genome segments or of pigs as mediators of infection in the generation of the human pandemic viruses of 1918, 1957 or 1968 since the origin of reassorted segments in those pandemic viruses have all been traced to avian hosts [88]. However, the initial host species in which the pandemic avian-human IAV reassortment occurred remains elusive, and very little surveillance for IAV in swine populations has been carried out at that time.

## Sustained avian IAV infection in pigs remains a rare event

Spillover infections of IAV of either human or avian origin into swine populations have been documented frequently in the past. Wild aquatic waterfowl are the reservoir of genetically diverse IAV. In fact, the highest variability in terms of hemagglutinin (HA) and neuraminidase (NA) subtypes of IAV is found in this reservoir [89, 90]. In general, IAV are host species restricted, however, some avian IAV subtypes are able to cross into non-avian species including pigs and humans [91]. Wholly avian IAV (AIV) of several subtypes have been isolated from pigs due to natural infection and pigs have also successfully been experimentally infected with a number of avian-origin IAV subtypes (Table 2 (20)). For example, avian IAV of subtypes H4N6 and H6N6 have been isolated from Canadian swine, also, H4N6 was detected in the United States, all with no sign of onward transmissions or adaptation to the swine population [92, 93]. In Asia, a wide range of subtypes has been found in pigs (H3N2, H4N1, H4N8, H5N1, H6N6, H7N2, H9N2, H10N5) but these also did not fully adapt to swine and resulted in dead-end infections [94–101]. Likewise, attempts to adapt avian IAV of the H9N2 subtype to swine in inoculation experiments and forced consecutive passaging enhanced replication and transmission of the virus but did not result in full adaptation [102].

**Table 2:** Sporadic infections in pigs with influenza A viruses of avian origin

Continent	Country	Subtype	Year	References
North America	Canada	H4N6	1999	[92]
		H3N3	2001	[103]
		H1N1	2002	[103]
	United States	H4N6	2015	[93]
Asia	China	H9N2	1998–2007	[94, 94, 104]
		H7N2	2001	[95]
		H10N5	2008	[96]
		H5N1	2008–2009	[97]
		H4N1	2009	[101]
		H6N6	2010	[99]
		H3N2	2011	[100]
		H4N8	2011	[101]
	Indonesia	H5N1	2005–2007	[105]
	Korea	H5N2	2008	[106]
Europe	Belgium*	H1N1	1979	[17, 107]
	England	H1N7	1992	[108]

*First detected in Belgium, H1avN1 spread rapidly through other European countries

An important exception is the Eurasian avian-like swine H1N1 lineage, which emerged in swine in Belgium and Germany in the 1970s and was closely related to a H1N1 virus isolated at that time from wild ducks. However, this incidence is thought to be the first evidence of a direct spill-over of an avian IAV into swine [17, 109]. It rapidly spread through European countries, replaced the previously circulating classical H1N1 swine lineage and became enzootic. Reassortment events with seasonal human H3N2 in the 1980s and H1N1 in the 1990s led to the new, stably circulating swIAV lineages, comprising gene segments of avian, swine and human origin [109].

## Reverse zoonotic infections of swine with human IAV occur frequently and drive the emergence and evolution of swine-adapted lineages

The most commonly detected swIAV circulating in pig populations around the globe are of subtypes H1N1, H1N2 and H3N2 [18, 110]. The first documented introduction of human IAV into swine populations occurred in the aftermath of the Spanish flu; this lineage was designated “classical swine” H1N1 (or lineage 1A). Thereafter, the genetic diversity of swIAV has grossly extended due to further incursions of human-derived pandemic and seasonal IAV [5, 14, 111]. In Europe, avian-derived IAV have also contributed to the diversity of swIAV. Around the globe, further reassortments and genetic drift have led to the circulation of highly divergent swIAV lineages [112]. One example is the triple reassortant swIAV (TRIG), which evolved in North America in 1998. Often, several subtypes are co-circulating and fluctuate in relative prevalence regionally. Nelson et al. [111] and Karasin et al. [113] identified swine IAV of the subtype H3N2 in North America which possess without exception all segments of a human IAV and had been circulating undetected in the swine population for several years. In Denmark, swIAV reassortants of the H3N2 subtype were detected in 2013 that derived from human seasonal H3N2 strains of the 2004/5 season [114]. This again suggests the sustained but undetected circulation of human IAV (or parts thereof) in swine populations indicating that pigs may serve as reservoirs of “old” human IAV long after these viruses have ceased to circulate in human populations: Souza et al. [25] identified swIAV H3 lineages in North American pigs that were antigenically distinct from seasonal human H3 vaccine strains currently used in the US. These swine H3N2 lineages originated from human sources in the 1990s and 2010s, and have been circulating enzootically in swine populations in the US until today. While human H3N2 viruses have undergone substantial antigenic drift since 1990, the swine viruses retained their close antigenic relation to the original human H3N2 strains. This type of "frozen evolution" in pig populations creates a gap to the current H3N2-specific immunity in the human population, particularly affecting people born after 1990. Therefore, current vaccines cannot induce adequate protective immunity in the human population against swIAV derived from older IAV of human origin. This results in an increased risk of zoonotic spillover events [25, 33].

The pandemic virus H1N1pdm09 was a reassortant of the TRIG, Eurasian-avian and the classical swine H1N1 lineage [7, 112]. This virus notably seemed to prove the “mixing vessel” hypothesis and the threat of pigs generating zoonotic IAV. The origin of the pandemic strain has been traced back to swine populations in central Mesoamerica [75]. Starting already in 2009 and continuing up to date, frequent reverse zoonotic transmissions of H1N1pdm09 into swine populations have been a major factor in the recently increasing genetic diversity of swIAV worldwide. Repeated introductions of seasonal as well as pandemic IAV of human origin since 1918 significantly contributed to expand the genetic diversity of swIAV globally, also prior to the 2009 pandemic. These processes continue to generate a plethora of novel genotypes [112, 115]. In a European surveillance study, Henritzi et al. [18] identified emerging swIAV reassortants with enhanced zoonotic potential in European swine holdings, including at least 31 novel genotypes partially carrying gene segments that were derived from human H1N1pdm09 IAV.

Enzootic year-round swIAV circulation in commercial swine farms is another important driver in the ecology of zoonotic IAV [3, 112]. Such recently discovered and widely proliferating forms of self-sustaining modes of swIAV infections in large swine holdings challenge preventive concepts based on vaccination with licensed adjuvanted, inactivated swIAV vaccines by generating holding-specific vaccine-escape mutants in rolling circles of infection. The European research consortium PIGIE is currently examining details of such “persistently” infected swine holdings [116].

## The “poor pig” hypothesis: pig populations suffer more frequently from reverse zoonotic IAV infections than humans from zoonotic swIAV transmissions

**Fig. 2:**
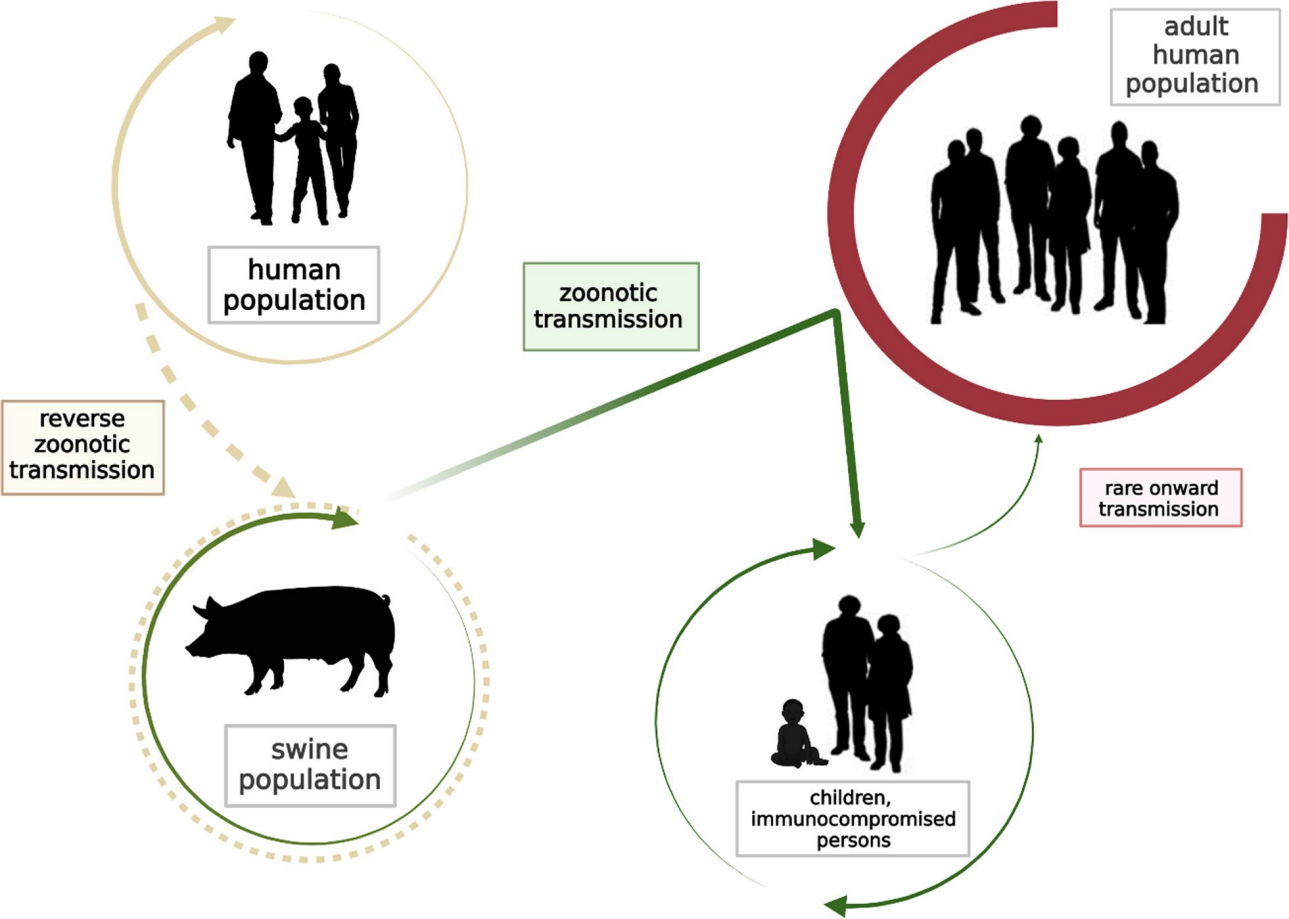
Proposed scheme of mutual transmissions of influenza A viruses (IAV) between human and porcine populations. Reverse zoonotic IAV transmission from humans to swine is a major driver of IAV diversity in pigs. “Historic” human IAV lineages may circulate for prolonged periods in pigs when their counterparts in humans have already been replaced; co-infections of such viruses in pigs with other IAV of porcine or avian origin may produce reassortants with enhanced zoonotic or even pre-pandemic potential. Zoonotic transmission back to the adult human population is probably sporadic and rare due to their substantial cross-reactive immunity (red barrier). Children and immunocompromised patients, in contrast, may have a higher susceptibility

A schematic overview of the flow of IAV between human and swine populations is provided in Fig. 2. There is no easy answer to the question why apparently more often IAV is transmitted from humans to pigs than vice versa. Receptor-bearing, permissive host cells in both species should be accessible with similar ease for viruses in the upper respiratory tracts.

Differences in population structures and population immunity of pigs and their keepers provide a possible first explanation: Adult staff working in swine holdings or having otherwise occupational exposure should have at least partial cross-immunity to different influenza subtypes due to previous exposure to human seasonal and/or pandemic IAV through multiple infections or vaccinations. In fact, the adult human population was shown to possess cross reactive antibodies in hemagglutinating and neutralizing assays against various swIAV subtypes [6, 18]. In contrast, the porcine population structure in modern production systems is extremely flat, and the majority of individuals consists of piglets which present an inexperienced immune system [6]. Maternal immunity passed on to the piglets via colostrum has been shown not to be effective in preventing suckling piglets from swIAV infection although they do not develop overt clinical signs [117, 118]. Despite early infection in life, the animals regain susceptibility to IAV infections after 6–12 weeks, in line with constant turn-over and the decline of maternal immunity. Thus, in intensive piglet-producing farms, a substantial part of the swine population is permanently available as susceptible hosts of IAV while the adult staff of such holdings likely refers to a much broader repertoire of adaptive IAV-directed immunity. This would pose a higher obstacle for swIAV to cross the human species border as compared to human IAV infecting newborn or juvenile pigs. In line with these thoughts, case reports of human infections with swIAV list a surprisingly high number of children, adolescents or immunocompromised patients (Table 1). This could signal a higher susceptibility to swIAV of the younger age sector of the human population due to their limited repertoire of cross-reactive IAV immunity. Thus, personnel in pig farms should receive annual vaccinations against seasonal influenza and staff with respiratory symptoms during the influenza season should avoid contact with pigs in order to reduce the risk of human-to-swine IAV transmission [119].

The high density of susceptible porcine individuals in large holdings might not only provide advantageous conditions for transmission and spread of swIAV but also of human-origin IAV that are not optimally adapted to pigs. Co-circulation of an optimally adapted porcine IAV with a newly introduced human IAV would provide reassortment opportunities that could foster further adaptation of the human IAV.

Furthermore, effectors of innate immunity, such as interferon-stimulated Mx1 proteins with anti-IAV activity, also have to be considered when looking at transmission events between human and swine populations. It has been well established that human Mx1 is a key factor in the species barrier preventing zoonotic IAV spill overs, especially from the avian reservoir [120]. Consequently, a prerequisite for all IAV to establish a new lineage and sustained circulation in the human population is the escape from human Mx1 restriction, a property found in all human, pandemic and seasonal IAV strains. Human-adapted IAV can also evade inhibition by porcine Mx1, which shows less potent antiviral activity compared to human Mx1, facilitating reverse zoonotic transmission into swine populations [121]. Due to its weaker activity, however, porcine Mx1 can promote preadaptation of IAV to human Mx1. Currently circulating swIAV have been detected that have already acquired full or partial resistance to human Mx1 [18, 122]. Interestingly, during reverse zoonotic transmission events human IAV lose some of the Mx1 resistance-conferring adaptations, since the escape from Mx1 is associated with a general fitness loss requiring compensatory mutations [121, 123].

## A plea for regulated, close-meshed IAV surveillance of domestic pig populations

The relationship of porcine and human populations with respect to mutual transmissions of IAV is complex. Swine populations reportedly maintain the circulation of swIAV with zoonotic and rarely (pre)pandemic potential. Thus, the importance of pig populations as a source of zoonotic IAV should not be underestimated. On the other hand, decades of intensive pig rearing have not produced frequent swine-to-human transmissions that resulted in new, sustained human IAV lineages. Recently, insight was gained into the capacity of other species, including humans themselves, to act as mixing vessels of IAV of different host origins. In addition, direct avian-to-human IAV transmission events have frequently been reported, in particular for high pathogenicity avian IAV associated with high case fatalities [124]. Thus, pig populations should not be globally stigmatized as the sole reservoir of potentially zoonotic IAV. The emergence of the most recent human IAV pandemic in 2009, however, has clearly demonstrated the principal risk of swine populations in which IAV circulate unimpededly. Therefore, the most important lesson to be learnt is to implement regular and close-meshed IAV surveillance of domestic swine populations to be able to follow the dynamics of swIAV evolution. The appropriate tools, such as real-time RT-PCR and next generation sequencing, are well established. However, improved algorithms for directly inferring zoonotic potential from whole genome sequences are still being sought to avoid human staff of swine holdings or visitors of agricultural fairs as involuntary sentinels for swIAV with increased zoonotic potential. Transboundary exchange of such data via shared databases would also facilitate the constant update and improvement of effective vaccines for swine as the most important preventive measure to reduce the viral load at the porcine-human interface. With regard to further improved risk assessment, it would be interesting to examine whether sera from children and adolescents who have had less exposure to IAV infections also show lower cross-reactive antibody titres and, hence, increased susceptibility to porcine IAV compared to adults.

## Data Availability

Data sharing is not applicable to this article as no new data were generated or analysed during the current study.

## Abbreviations

AIV: Avian influenza (A) virus
ANP32A: Acidic nuclear phosphoprotein 32 family member A
IAV: Influenza A virus
HA: Hemagglutinin
H1av: H1, avian-like or lineage 1C
HN1pdm2009: H1N1, human pandemic virus of 2009 or lineage 1A
H3hu: H3, human-like
H1hu: H1, human-like or lineage 1B
M: Matrix gene
NA: Neuraminidase
RT-PCR: Reverse transcriptase PCR
SA: Sialic acid
swIAV: Swine Influenza A virus
TRIG: Triple reassortant (internal gene) H3N2
“v”: Variant
